# Ultrasound Measurement of the Fetal Adrenal Gland and Prediction of Preterm Birth

**DOI:** 10.7759/cureus.101989

**Published:** 2026-01-21

**Authors:** Martiniuc Ana Elena, Toader Oana Daniela, Pop Lucian Gheorghe, Georgescu Tiberiu Augustin, Suciu Ioan Dumitru, Tigoianu Laura, Nicolae Suciu

**Affiliations:** 1 Obstetrics and Gynecology, Carol Davila University of Medicine and Pharmacy, National Institute for Mother and Child Health (INSMC) “Alfred Rusescu", Bucharest, ROU; 2 Pathology, Carol Davila University of Medicine and Pharmacy, National Institute for Mother and Child Health (INSMC) “Alfred Rusescu", Bucharest, ROU

**Keywords:** cervical length, corrected adrenal gland volume, fetal adrenal gland, preterm birth, ultrasound biometry d/d ratio

## Abstract

Introduction

Identifying a marker that is highly sensitive, specific, noninvasive, and cost-effective for predicting preterm labor remains a critical clinical priority. We propose that ultrasound assessment of fetal adrenal gland morphometry, especially measurement of adrenal gland volume, may help discriminate pregnancies at higher short-term risk of delivery among women presenting with threatened preterm labor.

Objective

The objective of the study is to evaluate the short-term discriminative performance of ultrasound-derived fetal adrenal gland morphometric parameters, particularly the fetal zone depth-to-total gland depth (d/D) ratio and corrected adrenal gland volume (cAGV), in identifying the risk of delivery within seven days in patients with threatened preterm labor.

Methods

This retrospective study included 52 singleton pregnancies between 28 and 35 weeks' gestation. Fetal adrenal gland dimensions and the fetal zone were measured via transabdominal ultrasound. Logistic regression and receiver operating characteristic (ROC) analysis were used to assess predictors of preterm birth within seven days of ultrasound evaluation.

Results

Both d/D ratio and cAGV were significantly higher in the early delivery group (p < 0.0001). Multivariable logistic regression identified d/D ratio as the strongest association with delivery within seven days. The model yielded an area under the curve (AUC) of 0.88. At the optimal probability threshold of 0.59, sensitivity was 85.7%, specificity 79.2%, positive predictive value (PPV) 82.8%, and negative predictive value (NPV) 83.3%.

Conclusion

Fetal adrenal morphometry, particularly d/D ratio and cAGV, can serve as valuable predictors of imminent preterm delivery and should be integrated into clinical assessment tools for symptomatic patients.

## Introduction

There is a clear and pressing need for a highly sensitive, specific, noninvasive, and cost-effective marker to predict preterm labor [1]. Preterm birth remains the leading cause of neonatal morbidity and mortality worldwide, accounting for substantial perinatal deaths and long-term complications in survivors [2]. Although transvaginal cervical length measurement is widely used for risk stratification, its diagnostic performance in symptomatic women is limited. Placental activation plays a central role in initiating the cascade of events that lead to labor [3-5].

Anatomy of the fetal adrenal gland

The fetal adrenal gland is a relatively large, triangular organ situated superior to the fetal kidneys and adjacent to the great vessels. Unlike the adult gland, which is composed mainly of cortex and medulla, the fetal adrenal gland has a distinct fetal zone that accounts for approximately 80%-90% of its total volume during mid-gestation.

Histologically, the fetal adrenal gland consists of three zones [6]: The definitive zone (outer cortex) is a thin layer that later gives rise to the adult adrenal cortex. The fetal zone (inner cortex) is the dominant compartment during gestation, characterized by rapid growth and high steroidogenic activity. It primarily produces dehydroepiandrosterone sulfate (DHEA-S), a precursor for placental estrogen synthesis [7]. The medulla, still underdeveloped in utero, matures postnatally and contributes minimally to fetal endocrine function.

The fetal zone regresses rapidly after birth, accounting for the marked reduction in adrenal size in neonates compared with fetuses. Its dynamic growth and regression reflect the close integration of the adrenal gland with placental and maternal hormonal pathways [6,8].

From an imaging perspective, the gland appears as a hypoechoic structure on ultrasound, often described as “V- or Y-shaped” above the renal poles. The fetal zone depth-to-total gland depth (d/D) ratio reflects the proportion of the gland occupied by the fetal zone, while the corrected adrenal gland volume (cAGV) normalizes overall gland size for fetal weight. Both parameters provide insight into endocrine activity and have emerged as promising biomarkers for imminent preterm birth [9].

As term approaches-or in certain pathological conditions-placenta increases secretion of corticotropin-releasing hormone (CRH), which stimulates the fetal hypothalamic-pituitary-adrenal (HPA) axis. This activation results in progressive maturation and functional changes within the fetal adrenal gland, particularly enlargement of the fetal zone and increased steroidogenic activity [10]. The gland produces higher levels of DHEA-S and cortisol, which are converted by the placenta into estrogens. Rising estrogen concentrations, in turn, enhance prostaglandin synthesis and receptor expression in the myometrium, promoting cervical ripening, membrane rupture, and effective uterine contractions [8]. In preterm labor, premature activation of this placenta-fetal adrenal axis can trigger these processes ahead of schedule, often in response to maternal or fetal stress, infection, or inflammation [11]. This interplay underscores the importance of fetal adrenal morphometry as a biomarker for identifying pregnancies at imminent risk of delivery [9,12,13]. Increasing evidence suggests that activation of the fetal HPA axis, reflected in morphological changes of the fetal adrenal gland, plays a pivotal role in the cascade leading to spontaneous labor. Sonographic assessment of fetal adrenal gland dimensions, particularly the d/D ratio and cAGV, has emerged as a promising approach to identify pregnancies at imminent risk.

Our primary objective was to evaluate the predictive performance of ultrasound-derived fetal adrenal gland morphometric parameters-specifically the d/D ratio and cAGV-for identifying delivery within seven days in women presenting with threatened preterm labor. Our secondary objectives were to assess whether combining fetal adrenal measurements with cervical length improves predictive accuracy and to compare fetal adrenal gland morphometry between pregnancies resulting in delivery within and beyond seven days using parametric and nonparametric statistical methods.

We hypothesize that ultrasound-based measurement of fetal adrenal gland morphometry reflects activation of endocrine pathways associated with impending labor and may serve as a phenotypic marker of short-term delivery risk in symptomatic pregnancies, rather than as an independent causal determinant of preterm birth [14]. This approach not only improves diagnostic accuracy but also enables more precise clinical stratification, ensuring that only women at genuine high risk receive medication or complex interventions, while avoiding unnecessary treatment in low-risk cases [3]. This is particularly relevant in our country, where preterm birth remains a major public health concern, neonatal intensive care resources are limited, and timely, targeted management can significantly improve perinatal outcomes [15,16].

## Materials and methods

This retrospective study was conducted at the Alessandrescu-Rusescu National Institute for Mother and Child Health, Bucharest, Romania, a tertiary-care referral hospital, between January and November 2025. The institution serves as a regional referral center for high-risk obstetric care, and as such, the study population may represent a higher-risk cohort than the general obstetric population.

All singleton pregnancies between 28 and 35 weeks’ gestation referred for evaluation of threatened preterm labor during the study period were consecutively included if they met the predefined inclusion criteria. Patients were excluded for multiple gestation, maternal comorbidities, or incomplete clinical data. This consecutive sampling strategy was used to reduce selection bias. Ultrasound evaluation of the fetal adrenal gland was performed at the time of clinical presentation for threatened preterm labor and prior to initiation of therapeutic interventions, including tocolytic therapy or antenatal corticosteroids, whenever feasible.

This retrospective analysis included 52 singleton pregnancies between 28 and 35 weeks of gestation referred for preterm labor assessment. A transabdominal 2D ultrasound was performed to measure fetal adrenal gland length (L), width (W), depth (D), and fetal zone depth (d). For each examination, length, width, and depth were measured for both the total fetal adrenal gland and the fetal zone using orthogonal imaging planes. These measurements were used to calculate adrenal gland volume and fetal zone metrics. Volumes were calculated using the ellipsoid formula: Volume = (π/6) × L × W × D. The cAGV was defined as AGV divided by estimated fetal weight (EFW). The d/D ratio was computed as the mean fetal zone depth divided by total gland depth.

The outcome of interest was delivery within seven days of ultrasound. Logistic regression was used to model predictors, and receiver operating characteristic (ROC) curve analysis was performed to assess discrimination [3,15].

**Figure 1:**
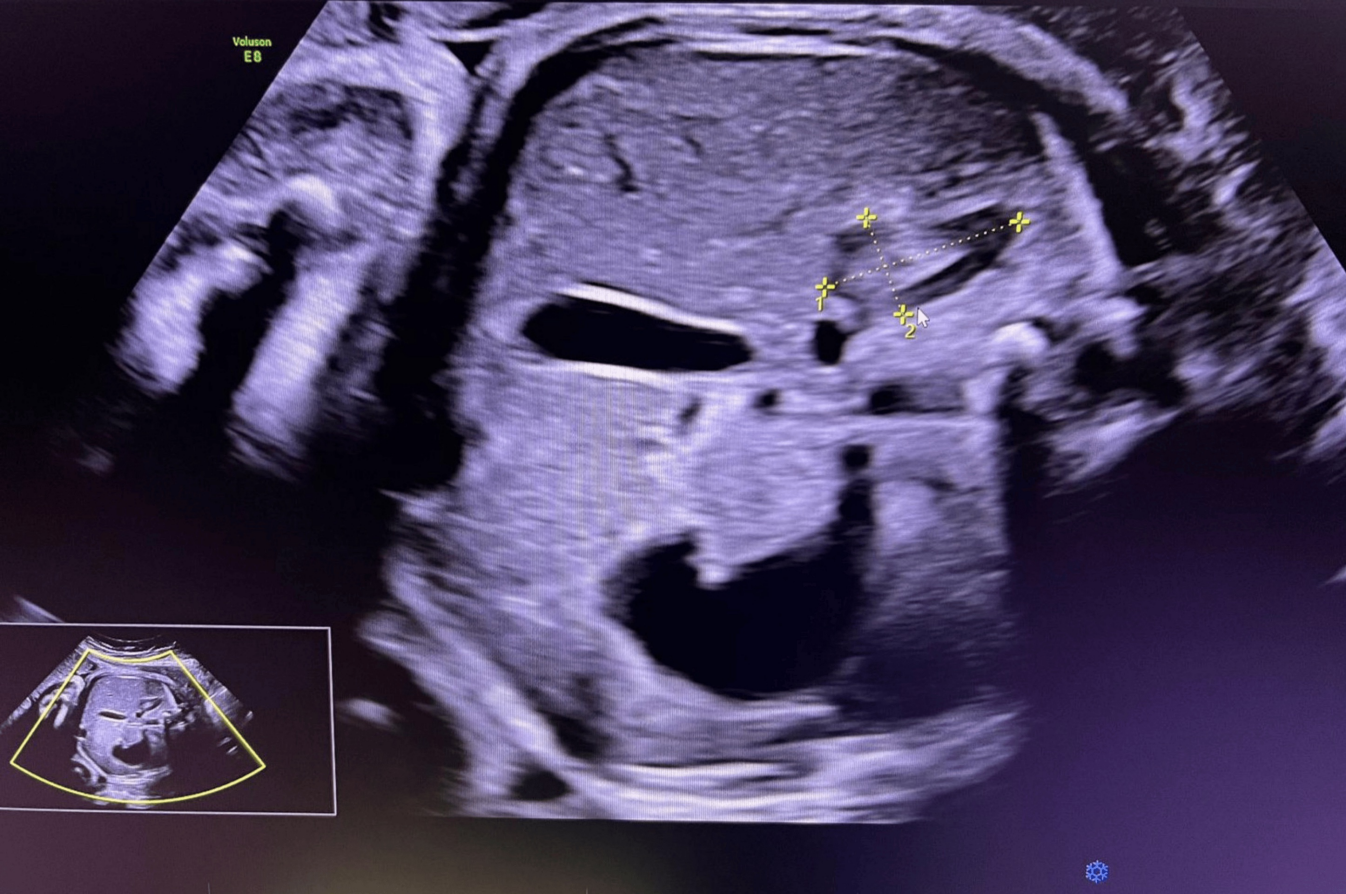
Axial ultrasound image of the fetal abdomen at 32 weeks’ gestation showing the right adrenal gland. The total gland length, width, and fetal zone dimensions are indicated

**Figure 2:**
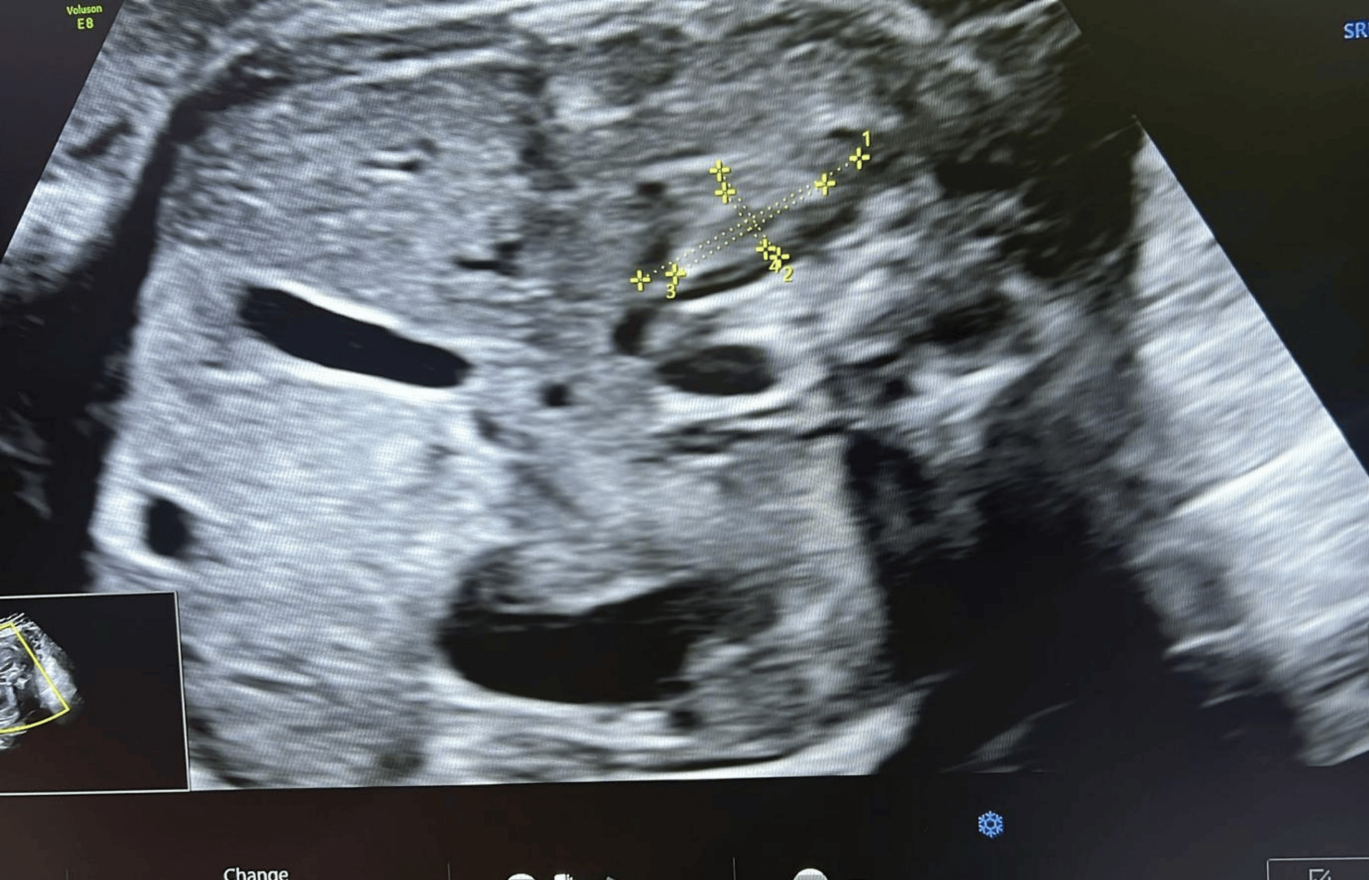
Image of the fetal adrenal gland demonstrating measurement of gland length and transverse dimensions using electronic calipers

**Figure 3:**
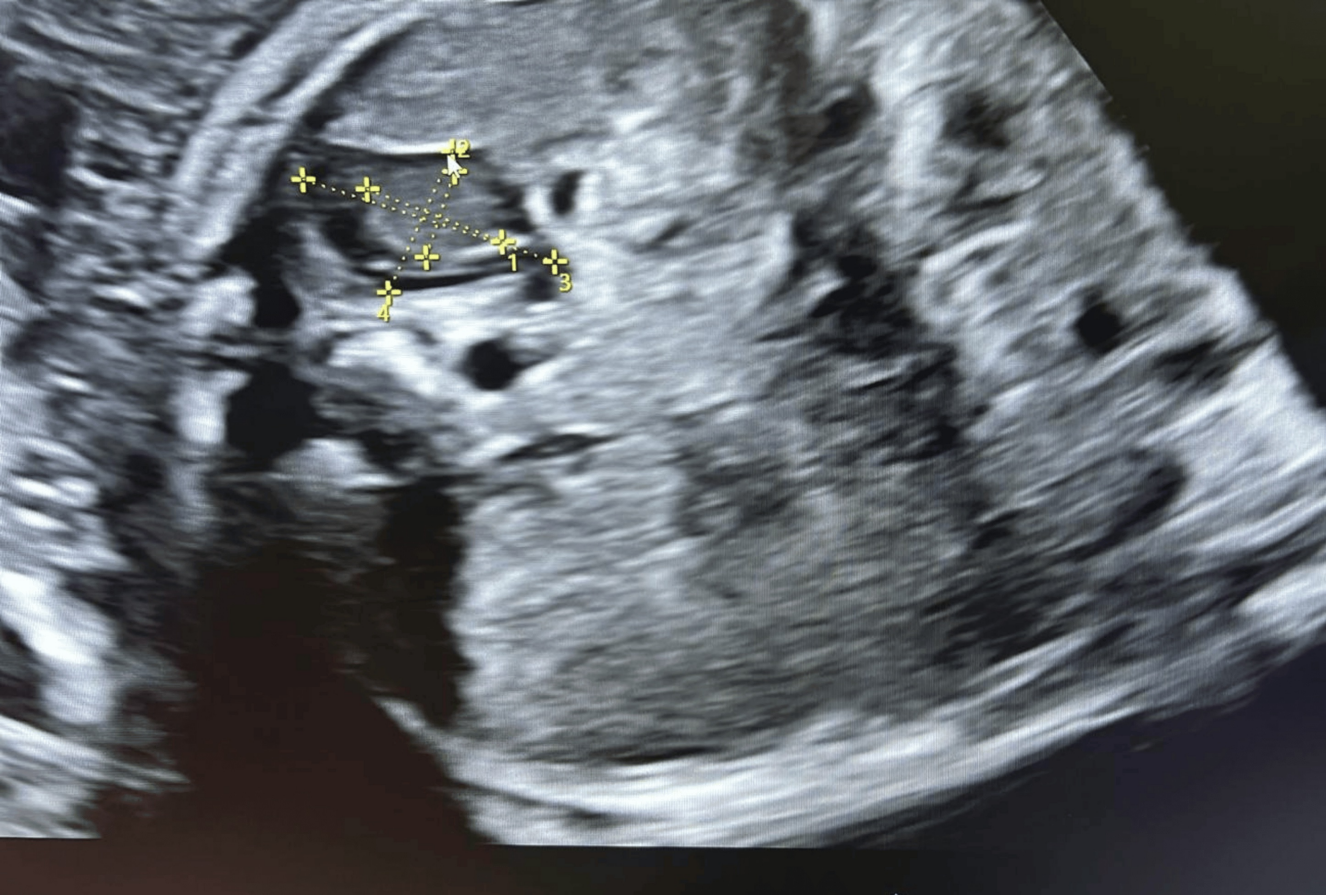
The gland exhibits its characteristic zonal architecture: a central echogenic medulla surrounded by a hypoechoic fetal zone and a thin peripheral definitive zone

The fetal adrenal gland was identified in the transverse abdominal plane just above the renal poles. Measurements were obtained from the fetal adrenal gland that was closest to the ultrasound transducer and most clearly visualized at the time of examination, irrespective of side. This pragmatic approach was adopted to optimize image quality and measurement accuracy in routine transabdominal scanning. The total gland length (L), width (W), and depth (D) as well as the fetal zone dimensions (l, w, d) were measured using electronic calipers (Figures 1-3). All measurements were performed by trained sonographers and reviewed by a maternal-fetal medicine specialist [17]. The d/D ratio was derived by dividing fetal zone depth (d) by total gland depth (D), representing the relative size of the fetal zone. All volume estimates were expressed in milliliters (mL). Measurements were recorded in triplicate and averaged to reduce variability [18]. Data were analyzed using Python (v3.11; Python Software Foundation, Fredericksburg, VA, US) with sklearn and scipy libraries. Univariate comparisons were performed using independent t-tests. Multivariate prediction modeling was conducted using penalized logistic regression with L2 regularization. Model performance was evaluated using ROC curve analysis, and optimal threshold selection was based on the Youden Index. Sensitivity, specificity, positive predictive value (PPV), and negative predictive value (NPV) were reported for the optimal model [19]. All assessment tools used in this study, including ultrasound biometry of the fetal adrenal gland (d/D ratio and cAGV), cervical length measurement, and logistic regression with ROC curve analysis are standard, widely used clinical and statistical methods that are freely available and do not require licensing.

## Results

**Figure 4:**
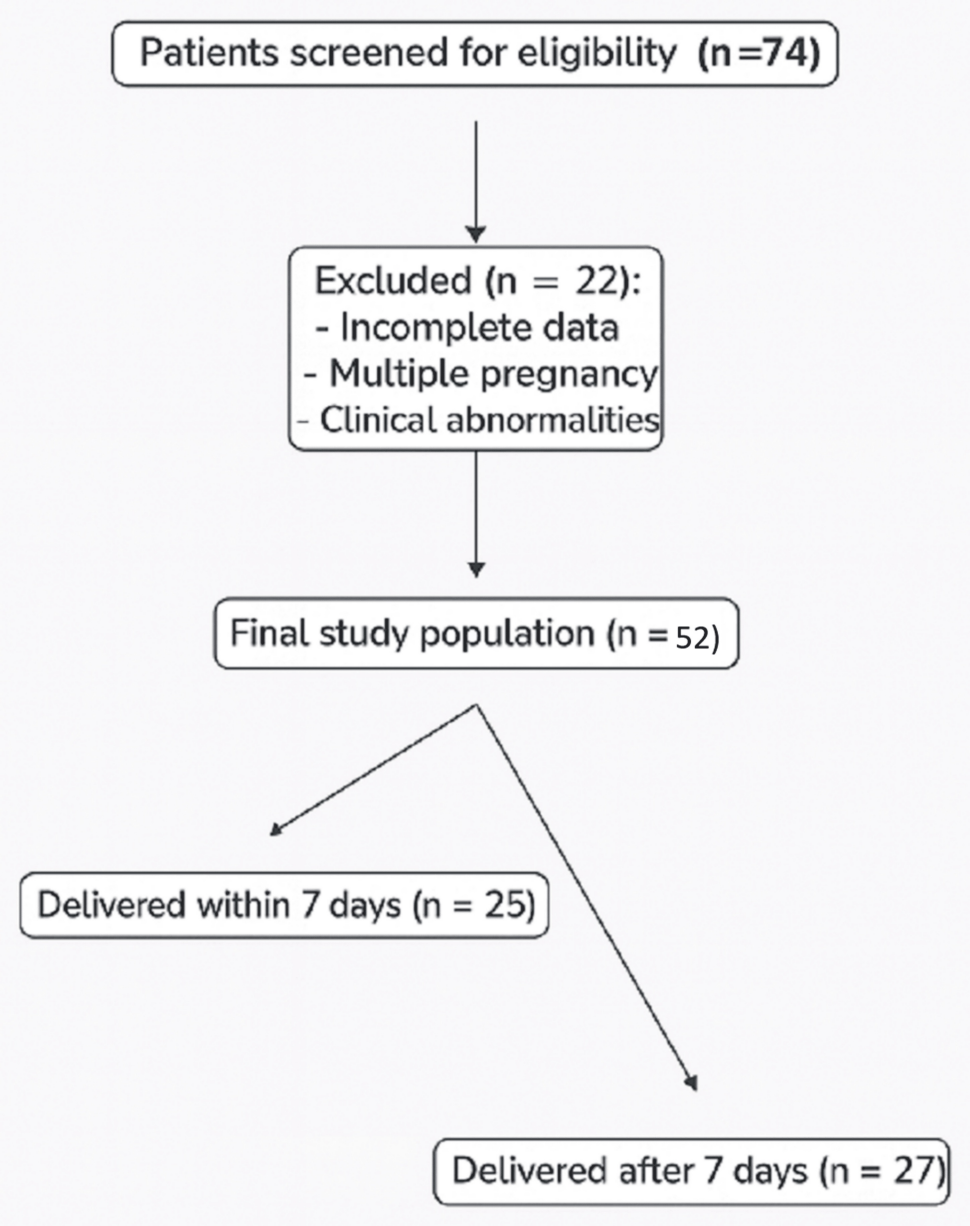
Flow diagram illustrating the selection of the study population, including initial recruitment, reasons for exclusion, and the final cohort of 52 singleton pregnancies analyzed

A total of 74 patients were initially recruited (Figure 4). Twenty-two were subsequently excluded: 10 because of incomplete pregnancy data, five due to multiple pregnancies, and seven owing to maternal comorbidities. Pregnancies complicated by fetal growth restriction or small-for-gestational-age fetuses were excluded to minimize potential confounding effects on fetal adrenal gland morphometry. Thus, 52 patients were included in the final analysis.

Out of 52 patients, 25 (48%) delivered within seven days. Baseline maternal and pregnancy characteristics of the study population, comparing patients who delivered within seven days to those who delivered after seven days from the ultrasound assessment, are presented (Table 1). The logistic regression model (Table 2) identified d/D ratio and cAGV as the most significant predictors of early delivery.

**Table 1:** Baseline characteristics of the study population (Mann-Whitney U test) GA: gestational age

Variable	≤7 days (n = 25)	>7 days (n = 27)	Mann–Whitney U	p-value
Maternal age (years)	28 (24.00–32.00)	31 (25.00–34.00)	300.0	0.4966
GA (weeks)	31 (28–35)	30 (29–32)	291.5	0.3998
Cervical length (mm)	21 (19.00–26.00)	29 (26.50–33.00)	75.0	0.00014

**Table 2:** Logistic regression coefficients (Wald test)

Predictor	Coefficient	Odds ratio
cAGV_calc	0.63	1.88
dD_ratio_calc	0.72	2.06
Cervical_Length	-1.80	0.17
GA	0.34	1.40
Maternal_Age	-0.29	0.75

In this cohort, the logistic regression model including d/D ratio, cAGV, and cervical length produced an area under the curve (AUC) of 0.941 (Table 2). The optimal decision threshold was 0.434, yielding 92.0% sensitivity and 88.9% specificity. The PPV and NPV were 88.5% and 91.4%, respectively. These findings demonstrate the strong predictive power of adrenal metrics and cervical length in symptomatic patients [5].

Additionally, univariate t-tests (Table 3) revealed statistically significant differences for d/D ratio, cAGV, and cervical length between those who delivered within seven days and those who did not. Mann-Whitney U tests further confirmed these findings, showing strong nonparametric evidence of distributional differences, especially for d/D ratio (U = 521.00), cAGV (U = 558.00), and cervical length (U = 75.00), all with p < 0.0001. No significant differences were observed for maternal age or gestational age in either test. These combined analyses underscore the robustness of the d/D ratio, cAGV, and cervical length as predictive markers of preterm birth in symptomatic pregnancies.

**Table 3:** Univariate comparison (t-tests) cAGV: corrected adrenal gland volume; d/D: fetal zone depth-to-total gland depth; GA: gestational age

Variable	≤7 days mean	>7 days mean	t-value	p-value
cAGV (mL/g)	0.00076	0.00054	t = 4.56	<0.0001
d/D ratio	0.561	0.498	t = 3.80	0.0004
Cervical length (mm)	22	30.26	t = −5.76	<0.0001
GA (weeks)	29.92	30.37	t = −0.74	0.4604
Maternal age (years)	28.88	29.85	t = −0.70	0.4861

In addition to parametric testing, nonparametric analysis using the Mann-Whitney U test (Table 4) further validated the predictive utility of key ultrasound-derived parameters. Both the cAGV and the d/D ratio demonstrated highly significant differences between women who delivered within seven days and those who did not (p < 0.0001), with distributions clearly shifted toward higher values in the preterm delivery group. Cervical length also differed significantly (U = 75.0, p < 0.0001), consistent with its well-established association with preterm labor [5,20]. In contrast, maternal age and gestational age showed no statistically significant distributional differences, reinforcing the specificity of adrenal and cervical measurements as key predictors of imminent delivery. These findings strengthen the robustness of the predictive model and support the clinical relevance of nonparametric evaluation in heterogeneous populations.

**Table 4:** Mann-Whitney U test results GA: gestational age

Variable	U statistic	p-value
cAGV_calc	558.00	p < 0.0001
dD_ratio_calc	521.00	p = 0.0008
Cervical_Length	75.00	p < 0.0001
GA	291.50	p = 0.3998
Maternal_Age	300.00	p = 0.4966

**Figure 5:**
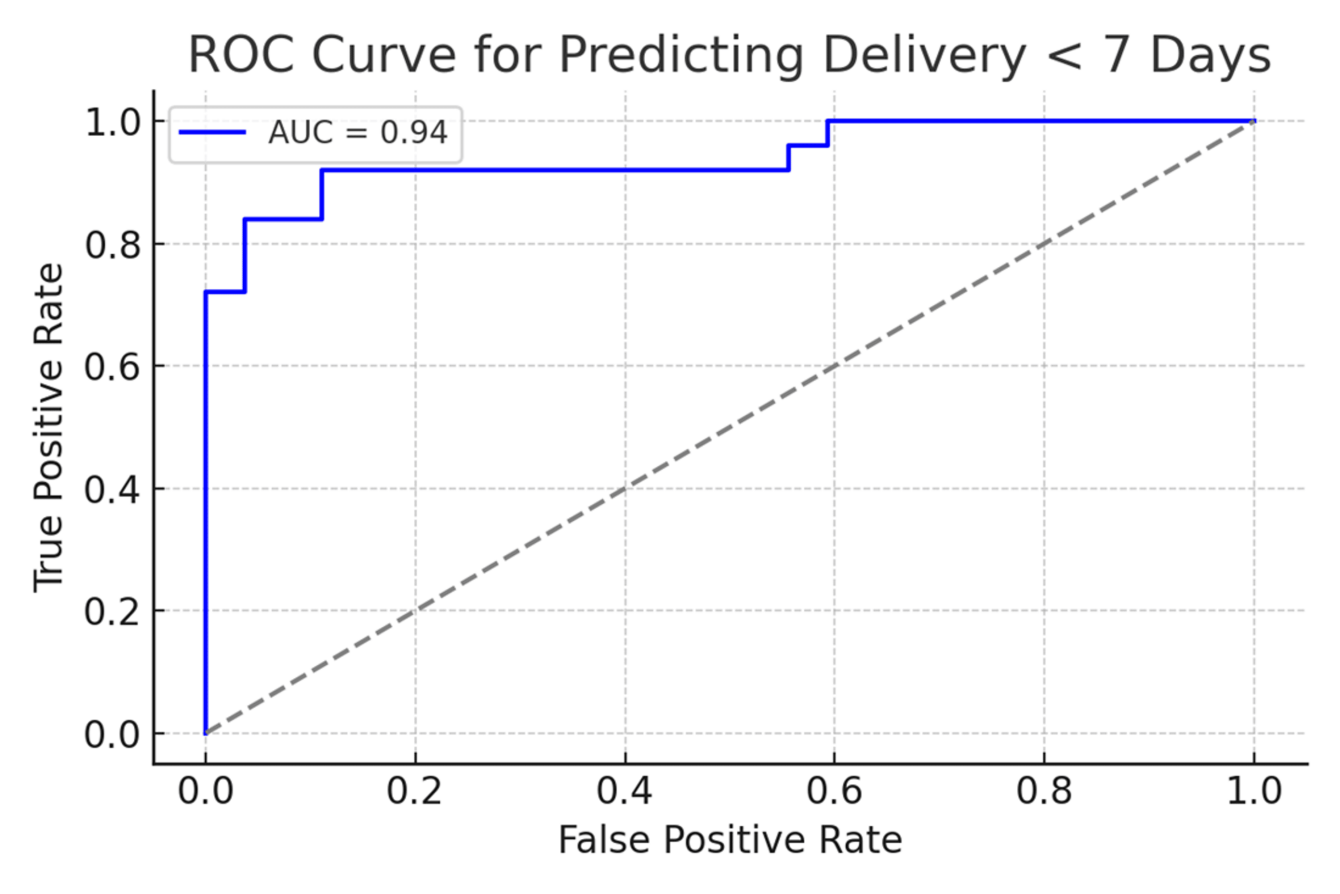
ROC curve ROC: receiver operating characteristic; AUC: area under the curve

The ROC curve analysis (Figure 5) demonstrated excellent model performance, with an AUC of 0.94. This high AUC indicates a strong ability of the model to discriminate between women who will deliver within seven days and those who will not. The optimal threshold identified using the Youden Index maximized both sensitivity (92.0%) and specificity (88.9%), ensuring reliable identification of high-risk cases while minimizing false positives. These findings support the clinical applicability of the combined model and suggest its potential utility in triage settings.

## Discussion

This study provides promising evidence that fetal adrenal gland morphometry is a highly effective predictor of imminent preterm delivery in symptomatic women between 28 and 35 weeks of gestation. Specifically, both the d/D ratio and cAGV demonstrated strong associations with delivery within seven days, reinforcing traditional sonographic markers such as cervical length.

The clinical relevance of these findings lies in the unique physiological role of the fetal adrenal gland in the initiation of labor. Premature activation of the fetal HPA axis increases production of DHEA-S and cortisol precursors that stimulate estrogen synthesis, membrane remodeling, and myometrial contractility, ultimately triggering the cascade toward labor. Therefore, increases in fetal adrenal gland size are not merely anatomical changes but reflect endocrine signaling that precedes spontaneous preterm birth [19,21].

The strong predictive performance of the logistic regression model (AUC 0.94), combined with high sensitivity (92.0%) and specificity (88.9%) at the optimal threshold, highlights the value of integrating adrenal sonography into clinical triage pathways. These metrics emphasize that adrenal biometrics can correctly identify the majority of patients who will deliver early, while minimizing unnecessary intervention among those likely to continue pregnancy.

Our findings are consistent with previous research by Turan et al. and Ibrahim et al., who demonstrated that adrenal gland enlargement is closely tied to imminent preterm delivery in symptomatic pregnancies. In contrast, Hoffman Sage et al. observed smaller adrenal volumes in asymptomatic women destined for spontaneous preterm birth. The difference likely reflects the timing and pathophysiology of true labor activation: in asymptomatic patients, preterm birth may result from inflammatory or structural pathways without fetal adrenal involvement, whereas in symptomatic patients, the fetal adrenal gland appears actively engaged in the transition toward delivery [3,21,22].

Importantly, the ultrasound techniques applied in this study rely on standard 2D measurements, making them practical for routine clinical use without requiring 3D or specialized imaging technology. The derived ratios and volume corrections reduce variability related to fetal size differences [22]. This enhances generalizability, particularly in resource-limited healthcare systems where preterm birth outcomes remain a major contributor to neonatal morbidity and mortality.

However, several limitations should be acknowledged. The retrospective design introduces the possibility of selection bias, and the modest sample size may restrict model precision and the ability to establish universal cutoff thresholds. The study population included only symptomatic women, limiting applicability for screening in the general obstetric population. Additionally, inter-observer variability in fetal adrenal gland measurements, though minimized through standardized methods, remains a challenge inherent to sonographic assessment. Formal inter-observer and intra-observer reproducibility analyses were not performed, which represents a limitation of this study. However, measurements were obtained by experienced operators, reviewed by a maternal-fetal medicine specialist, and averaged over three acquisitions to reduce measurement variability. Demographic and obstetric variables, including parity, race, and prior preterm birth, were not incorporated into the analysis; while these factors are relevant to overall preterm birth risk, the primary aim of this study was to assess the short-term predictive performance of fetal adrenal gland morphometry in symptomatic patients, independent of underlying etiology. Future prospective studies should incorporate comprehensive clinical, microbiological, and demographic data to further refine risk stratification.

Importantly, the observed associations between fetal adrenal gland morphometry and delivery within seven days should not be interpreted as evidence of an independent or causal relationship. Preterm birth is a multifactorial process influenced by infectious, inflammatory, obstetric, and demographic factors that were not fully captured in this retrospective analysis. In this context, adrenal gland enlargement is best interpreted as a phenotypic marker reflecting activation of endocrine pathways associated with impending labor rather than a sole determinant of outcome.

Future work should include multicenter prospective studies with larger and more diverse populations to validate threshold values, evaluate serial measurements, and explore integration with biochemical biomarkers of HPA axis activation. Machine learning models may further enhance risk prediction by incorporating clinical, hormonal, and sonographic variables into individualized probability estimates.

Overall, this study reinforces the fetal adrenal gland as a biologically meaningful and clinically powerful marker of imminent preterm birth. Incorporating d/D ratio and cAGV into assessment protocols for threatened preterm labor may substantially improve risk stratification, optimize treatment decisions, and reduce unnecessary interventions, ultimately improving neonatal outcomes.

## Conclusions

In symptomatic women at risk for preterm birth, ultrasound-derived fetal adrenal morphometry-particularly d/D ratio and cAGV-offers powerful predictive value for delivery within seven days. When combined with cervical length, these parameters enable high-accuracy risk stratification, allowing clinicians to identify which patients truly require medication or complex interventions, while sparing low-risk cases from unnecessary treatment. This targeted approach is of particular importance in our national healthcare setting, where optimizing resource allocation and preventing overtreatment can substantially reduce the burden of preterm birth.
